# Enabling Reliable UAV Control by Utilizing Multiple Protocols and Paths for Transmitting Duplicated Control Packets

**DOI:** 10.3390/s21093295

**Published:** 2021-05-10

**Authors:** Woonghee Lee

**Affiliations:** Division of IT Convergence Engineering, Hansung University, Seoul 02876, Korea; whlee@hansung.ac.kr; Tel.: +82-2-760-4225

**Keywords:** unmanned aerial vehicle, drone, control packet, multipath transmission control protocol, user datagram protocol

## Abstract

In the last ten years, supported by the advances in technologies for unmanned aerial vehicles (UAVs), UAVs have developed rapidly and are utilized for a wide range of applications. To operate UAVs safely, by exchanging control packets continuously, operators should be able to monitor UAVs in real-time and deal with any problems immediately. However, due to any networking problems or unstable wireless communications, control packets can be lost or transmissions can be delayed, which causes the unstable drone control. To overcome this limitation, in this paper, we propose *MuTran* for enabling reliable UAV control. *MuTran* considers the packet type and duplicates only control packets, not data packets. After that, *MuTran* transmits the original and duplicate packets through multiple protocols and paths to improve the reliability of control packet transmissions. We designed *MuTran* and conducted a lot of theoretical analyses to demonstrate the validity of *MuTran* and analyze it from various aspects. We implemented *MuTran* on real devices and evaluated *MuTran* using the devices. We conducted experiments to verify the limitations of the existing systems and demonstrate that control packets can be transmitted more stably by using *MuTran*. Through the analysis and experimental results, we confirmed that *MuTran* reduces the control packet transfer delay, which improves the reliability and stability of controlling UAVs.

## 1. Introduction

In recent years, technologies for unmanned aerial vehicles (UAVs) have developed considerably and UAVs, also known as drones, have recently received tremendous attention from many researchers [1,2]. Many service providers in various fields have also tried to utilize UAVs for their services [3]. UAVs can move freely in three dimensions and perform various operations to accomplish given missions from operators [4]. During the missions, UAVs constantly send and receive control packets with the operators, so that the operators continue to monitor the status of UAVs and control their behavior appropriately. Thus, delayed transmissions or losses of control packets can cause very dangerous situations although the size of control packets are generally much smaller than that of data packets. For example, in the process of controlling UAVs in real time, delayed transmissions or losses of control packets can cause catastrophic accidents, such as crashes or collisions with obstacles or other UAVs. Therefore, only when control packets are delivered at an appropriate time without loss or delay, UAVs perform their missions well.

Today, it is not difficult to utilize multiple communication modules together. Many studies have been conducted that utilize multiple interfaces simultaneously, including studies on improving the existing transmission control protocol (TCP) and user datagram protocol (UDP) for multipath transmissions [5]. If the state of one link deteriorates, by leveraging multiple paths, the user is able to continuously send and receive packets over the other paths, which improves the reliability and stability of data communications. However, these methods are not effective in preventing delays or losses of packets that have already been queued or sent because such methods are reactive approaches to address the issues. In other words, even if drones or users using these methods respond quickly to problems, it is inevitable that packets already transmitted might be delayed or lost due to the problems. Such delayed transmission or loss of control packets can be extremely fatal to fast-moving UAVs.

In this paper, we propose a new control packet transmission scheme for controlling UAVs, *MuTran*, to overcome the aforementioned problem. The existing MPTCP does not consider characteristics of packets when selecting a subflow for transmission [6]. In contrast, *MuTran* selectively duplicates only control packets, not data packets, considering the contents of the packets. Then, *MuTran* transfers the originals and their duplicates to different networks respectively using multiple protocols and paths. Through such behaviors, *MuTran* prevents delayed transmissions and losses of control packets, which improves the reliability of controlling UAVs. We designed *MuTran* and implemented it on real devices. We conducted various experiments to prove the effectiveness of *MuTran*, and we also performed theoretical analyses to prove *MuTran*’s validity. Furthermore, we analyzed *MuTran* from various perspectives, such as the number of subflows to guarantee the target latency, the overhead due to duplication, and the control packet transmission rate when using *MuTran*.

To summarize the contributions of this paper:

- We propose *MuTran* which reduces the control packet transfer delay, so UAVs can be controlled more reliably by using *MuTran*.
- We come up with a technique that selectively duplicates only control packets, not data packets, and transfers the originals and copies of them through different paths.
- We design *MuTran* to be able to utilize multiple protocols for transmitting data and control packets separately, so the control packets can be transferred to UAVs with guaranteed low-latency regardless of data traffic.
- We implemented *MuTran* on real devices and demonstrated its value by conducting many experiments using the devices.
- We demonstrate the validity of *MuTran* by theoretically analyzing it from various perspectives.

Drones are the most representative and widely used UAVs today, so we have focused on drones among various UAVs in this paper. However, since *MuTran* is not designed to be specific to a particular UAVs, *MuTran* can be applied to any UAVs.

The remainder of this paper is organized as follows: We introduce related work and describe *MuTran*’s novelties and advantages against the related work in Section 2. We explain the problem statement and describe the design and implementation details of the *MuTran* in Section 3. We perform various theoretical analyses of *MuTran* in Section 4. We describe the experiments we have conducted and evaluate *MuTran*’s performance in Section 5. We discuss several issues that need to be addressed further in Section 6. Finally, Section 7 concludes this paper.

## 2. Related Work

We introduce various studies related to our work and then describe *MuTran*’s novelties and advantages in comparison to the relevant studies in this section.

Since UAVs can fly fast and move freely in any direction, delays in control packet transmission can cause difficulty in controlling them properly. Therefore, many studies relevant to controlling UAVs focused on reducing control packet transfer delay to guarantee deterministic properties in packet delivery, positioning, navigation, and timing. Some of these studies tried to reduce delays in terms of the physical layer or media access control (MAC) layer. Firstly, Chandhar et al. proposed a research which reduces the communication interference using beamforming of a multiple input multiple output (MIMO), which made a significant improvement in delay reduction [7]. Ruan et al. proposes an adaptive channel division MAC protocol for UAV networks [8]. The proposed protocol flexibly adjusts parameters related to MAC according to the number of UAVs and traffic load to make full use of the channel resources and guarantee the communication performance of multiple UAVs. In addition to these studies, there are studies that focused on approaches in the transport layer or application layer. Dai et al. designed a relay mechanism based on software defined network (SDN) architecture [9]. By considering the network environment changes, the proposed architecture switches to MANET mode to guarantee UAVs’ carrier transmissions, so as to reduce the transmission delays of UAVs. Zhu et al. proposed a low latency clustering method for large scale drone swarms [10]. The authors established a model which solves the relationship between the number of drones and the number of cluster heads. By using the model, the communication delay can be minimized by obtaining the optimal number of drones and cluster heads.

The efficient routing for low packet transmission delays is essential when operating multiple drones. Therefore, some researchers conducted research to devise routing algorithms suitable for networks composed of UAVs. Bousbaa et al. proposed GeoUAVs which is a geocast routing protocol for a fleet of UAVs [11]. This protocol reduces the average transmission delay by delivering information to a specific group of UAVs identified by their geographical location. Zhang et al. proposed a low latency routing algorithm for UAV swam networks [12]. The algorithm utilizes the information about UAVs’ location and connectivity of the network to decrease the link average delay. Hong et al. proposed a proactive topology-aware routing scheme, which keeps tracking the mobility and topology changes [13]. According to the results, their proposed scheme is able to reduce the average delay by adapting routing quickly to topology changes.

Due to advances in communication and network technologies, it is not difficult for devices to transmit packets through multiple paths by leveraging multiple interfaces simultaneously. The communication reliability and throughput can be improved by transmitting packets over different paths, so some researchers tried to apply multi-path communications to drones. Chiba et al. proposed an application layer protocol that copies all packets and transmits them over two different paths [14]. The authors showed that the proposed protocol reduces the transmission delay and rate of packet loss through simulations. However, this protocol copies all packets without any consideration on characteristics of packets. Thus, this protocol unnecessarily wastes communication bandwidth, and it does not improve throughput despite utilizing multiple interfaces. To improve [14], the proposed scheme in [15] replicates control packets only. However, like [14], data and control packets are inserted into the same network queue competitively. Thus, diverse causes, such as the increase in the amount of data packets or data packet retransmissions, increase the network queuing delay, which lengthens the latency of control packet transmission. Thirdly, Shailendra et al. proposed a new path scheduler of multipath TCP (MPTCP), which distinguishes control packets from data packets [16]. After distinguishing, the proposed scheduler assigns data packets and control packets separately to two different interfaces for more reliable transmission of control packets. However, since the interface for control packet is only used to transmit control packets, the throughput of the data packet transmission is not enhanced even when the interface for the control packet is not used. Moreover, this scheduler does not fully utilize the communication bandwidth of the interface for control packets. This is because control packets are generally generated less frequently and are smaller in size compared to data packets.

*MuTran* has novelty and advantages in some respects compared to the above related studies. First, *MuTran* considers the contents of packets and then selectively copies only the control packets, not the data packets. *MuTran* then transfers the originals and copies through different paths to reduce the delay in delivering the control packets. Secondly, *MuTran* uses multiple interfaces fully to transmit data packets and control packets, so the use of *MuTran* increases the overall throughput. Thirdly, *MuTran* is able to utilize not only TCP but also UDP, so *MuTran* can transmit control packets through UDP connections separated by TCP connections used for data transmissions. Thus, the control packets can be transferred to UAVs with a guaranteed low-latency, regardless of large traffic or retransmissions of data packets. Fourth, unlike many related studies, which conducted performance evaluations only through simulations, we implemented *MuTran* in Linux kernel layer and conducted many experiments using real devices with *MuTran*. Finally, as well as evaluations through real-world experiments, we perform theoretical analyses to prove *MuTran*’s validity. In contrast, most related studies performed only experiments or simulations without theoretical analysis.

## 3. System Design and Implementation

In this section, we describe the problem statement and explain the concept of the techniques used in *MuTran*. Then, we give a detailed description of *MuTran*’s design and three main elements.

### 3.1. Problem Statement

When operating drones, an operator continuously exchanges control packets and data packets with the drones through wireless communications. In general, data packets contain video, audio, sensor data, processed data, and so forth. On the other hand, control packets usually include commands, such as mission requests and responses, various instructions, and so forth, and control information, such as heartbeat, system status, position information, neighborhood discovery, configuration, fleet management, and so forth, for UAV systems and networks [17]. Compared to the wired communication, the wireless communication is more unstable and its available bandwidth changes more frequently. In addition to this, the high mobility of drones further exacerbates the communication instability. If the quality of communication for drones becomes poor, packet transmissions can be severely delayed or packets can be lost, which destabilizes the drones’ flight. In particular, control packets should be delivered in time without loss or delay, so that the operator keeps monitoring the drones’ conditions and properly controls their operations. For example, a drone should receive a control packet every 500 ms at the latest when using the offboard mode of Pixhawk 4 (PX4) autopilot with MAVLink protocol. (The offboard mode is mainly used to control movement and posture of vehicles [18], and Pixhawk autopilot [19] and MAVLink [17] are one of the most widely used autopilots and protocols to communicate with small unmanned vehicles, respectively.) Therefore, problems with transferring control packets can cause serious accidents.

Utilizing MPTCP and multiple interfaces, even when the condition of one communication path deteriorates, the operator can communicate reliably with a drone over another communication path. However, this method is a reactive method to deal with these problems, so it is not effective in preventing the loss or delay of packets that have already been queued or transmitted. Even if control packets are not delivered to a drone for a very short period of time, such momentary problems of transmission can be serious. For example, a quadcopter flying at a speed of 30 m/s travels 3 m in just 100 ms. (30 m/s is the typical flight speed of a drone for disaster management [20].) Thus, the high reliability with short delay is the most important concern when operating drones.

### 3.2. Duplicated Control Packet Transmission

**Figure 1:**
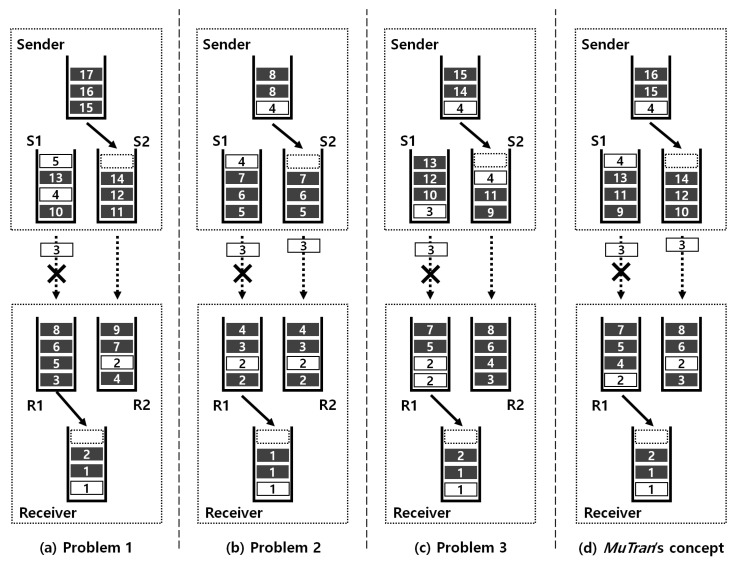
The packet transmissions of various cases. (This figure is drawn to distinctly present the difference between cases, so the ratio of control packets to data packets is exaggerated.)

Control packets are created less and are relatively small compared to data packets, but the control packet transmission with short delay is very essential for the reliable control of drones. Existing MPTCP-enabled systems do not prevent loss or delay of packets that have already been queued or sent as shown in Figure 1a. In the figure, there is one sender and one receiver, each with two wireless communication interfaces. The interfaces of the sender and the receiver are all identical, and each interface of the sender is connected to the receiver’s corresponding interface. The sender and receiver communicate via two paths, and these communication pairs use different channels to avoid interference. White blocks and black blocks mean control packets and data packets, respectively. In this situation, if the quality of the communication link on the left path deteriorates, control packet number 3 may be lost. In addition, all packets queued in the first subflow, including control packets number 4 and 5, can be delayed or lost. To overcome such a limitation, if all packets are copied without considering characteristics of packets as shown in Figure 1b, control packet number 3 is transferred successfully. However, the communication bandwidth is unnecessarily wasted, and the throughput cannot be improved even with multiple interfaces.

In existing systems with MPTCP, when a packet is inserted into one of the subflows, the default path scheduler selects the subflow with the shortest smoothed round trip time (SRTT). Therefore, the device cannot designate a specific subflow to transmit a certain packet. Thus, if the device using an existing system duplicates a packet and transmits the original and the duplicate, the both packets can be inserted into the same subflow as shown in Figure 1c. The duplicated packet is the following packet of the original, so the time between the insertions of them is too short for the communication situation to be changed. For this reason, using the existing system with MPTCP, the original and its duplicate are almost always inserted into the same subflow. If the original and the duplicate are transferred to the same path, it does not prevent delay or loss of control packets, and it also wastes the communication bandwidth unnecessarily. Therefore, the original and the duplicate should be transmitted through different paths.

In addition to the aforementioned scheduler using SRTT, by default, the MPTCP contains another scheduler, which transmits packets in a round-robin fashion. Therefore, if the device uses this scheduler to transfer the original and the duplicate, they can be transferred through different paths. This is the simplest way to transfer the original and the duplicate through different subflows. However, devices using this method transmit packets without considering the communication status of paths because packets are simply transmitted in a round-robin fashion. Therefore, in this case, the overall throughput can be degraded compared to the case when using the default scheduler utilizing SRTT. Section 5.2 shows this degradation.

To overcome the limitations mentioned above, we devise *MuTran*, which duplicates control packets and transfers the original and their duplicates through different paths and multiple protocols. The Figure 1d briefly shows the *MuTran*’s concept. As shown in the figure, even if one of the links suddenly becomes unavailable, control packet number 3 is transferred successfully.

### 3.3. Design of MuTran

**Figure 2:**
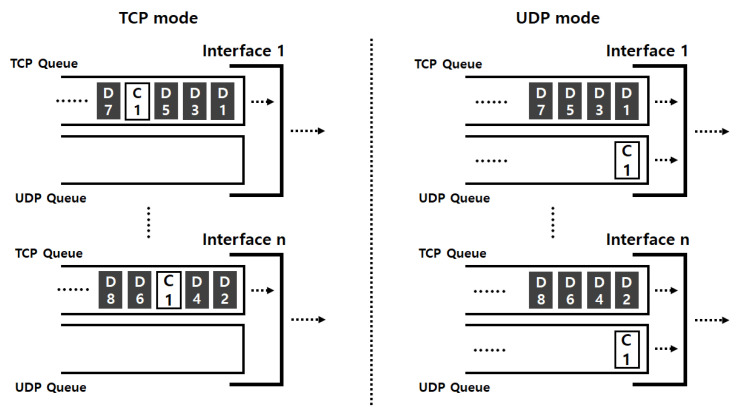
The difference between TCP and UDP modes of *MuTran*.

TCP and UDP are widely used transport protocols for UAVs, and they have different characteristics. TCP is a reliable transport protocol, so packet deliveries are guaranteed by using TCP. However, TCP has rather complicated operations, which causes a long transmission delay. Unlike TCP, UDP does not guarantee the delivery of all packets, but it is easier to implement UDP compared to TCP. In addition, UDP has a relatively high transmission rate because of fast processing with small overhead. Because of such advantages, UDP is more preferred by some platforms and protocols for UAVs, such as MAVlink. (MAVLink is a protocol dedicated for communicating with small unmanned vehicles, and MAVLink is widely used for commercial UAVs [17]. MAVLink mainly utilizes UDP and uses response messages for the sake of communication reliability [21].) However, many technologies support the both protocols because they have different strengths and weaknesses. For example, for shorter distance, UDP is better due to high throughput performance, whereas the connection-oriented protocol TCP is better for longer distance [22]. Thus, we design *MuTran* to be able to transmit control packets in two modes, TCP-mode and UDP-mode. In TCP-mode, not only data packets but also control packets are transferred by using MPTCP. In comparison, *MuTran* in UDP-mode transfers control packets using UDP, separated by transmitting data packets through MPTCP. Note that *MuTran* in the both modes transmits data packets using MPTCP for performance improvement and transfers original control packets and their duplicates through different paths for improving reliability. The difference between the two modes exists in protocols used for transmitting control packets as shown in Figure 2.

**Figure 3:**
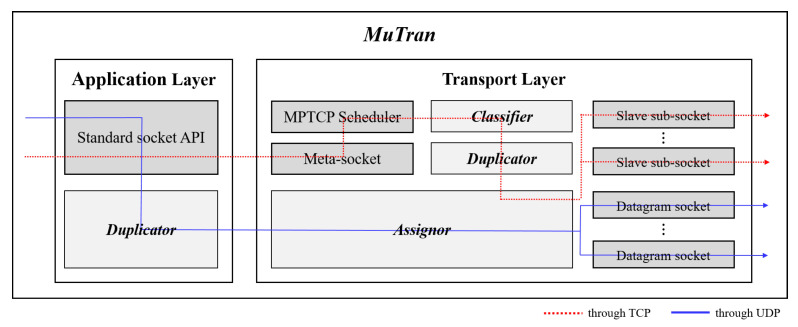
The structure and operation flow of *MuTran*.

Figure 3 shows the structure and operation flow of *MuTran*. The red-dotted line indicates the operation flow for transmitting control packets in TCP-mode, and the blue line with a relatively simple shape shows the operation flow in UDP-mode of *MuTran*. As shown in the figure, *MuTran* in UDP-mode operates more simply in comparison with *MuTran* in TCP-mode because of TCP’s innate complicated operations. Thus, in this subsection, we describe the design of *MuTran* with focusing on the TCP-mode and give additional explanations for understanding differences between operations in the TCP-mode and UDP-mode.

#### 3.3.1. *Mutran* with MPTCP

There are two types of socket, meta-socket and sub-socket, when using MPTCP as shown in Figure 3. To perform data communications, the application creates a meta-socket first and adds a sub-socket repeatedly as the application needs, so there are usually one meta-socket and multiple sub-sockets. The firstly created sub-socket among sub-sockets is called the master sub-socket, and the others are slave sub-sockets. When the application sends data using the standard socket application programming interface (API), the meta-socket receives the data and performs segmentation using the data. The meta socket then passes segments to the MPTCP scheduler. The MPTCP scheduler is responsible for assigning the segments to sub-sockets according to the rule, such as the shortest SRTT or round-robin. *MuTran* is made up of three main elements, *Classifier*, *Duplicator*, and *Assignor*, and we implemented these elements in the application layer and transport layer as shown in Figure 3.

#### 3.3.2. *Classifier*

**Figure 4:**
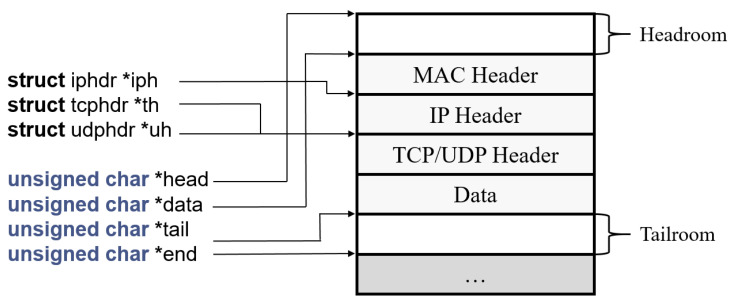
Pointers related to segment in kernel.

As the name suggests, *Classifier* has the role of classifying segments by data type. It is not trivial to know which data contains control information in the kernel layer. Among pointers related to segment in Linux kernel in Figure 4 [23], *Classifier* uses the pointer that points to the starting position of segment data in memory for direct access to the data. *Classifier* analyzes the directly accessed data to determine whether this segment has control information. If so, *Classifier* delivers the segment to *Duplicator*, otherwise *Classifier* does nothing. In UDP-mode, control packets and data packets are transmitted separately, so *Classifier* is not included in the operations as shown in Figure 3.

#### 3.3.3. *Duplicator*

*Duplicator* duplicates the segment received from *Classifier* and then forwards the original and the duplicates to the *Assignor*. *Duplicator* does not need to be located in the kernel layer, and it is possible for the application to contain *Duplicator* alternatively. In this case, the application knows whether or not a certain data has control information, so the *Duplicator* can only duplicate control data without *Classifier*. *MuTran* in UDP-mode does not use MPTCP to transmit control packets, so it is unnecessary to duplicate control packets in the kernel layer. Thus, as shown in Figure 3, *Duplicator* in the application layer duplicates control packets when *MuTran* operates in UDP-mode.

#### 3.3.4. *Assignor*

*Assignor* has the role of distributing the original and the duplicates to sub-sockets belonging to different interfaces. First, *Assignor* delivers the original to the most preferred sub-socket based on the default scheduler’s assessment. *Assignor* then selects the most preferred sub-socket of each interface that is not associated with the sub-socket used to transmit the original packet. *Assignor* transmits the duplicated packets through each of the different interfaces using the selected sub-sockets. In the UDP-mode, each interface has one datagram socket for UDP, so *Assignor* simply distributes the original and its duplicates to a socket of each interface.

After the sender with *MuTran* transmits the original and its duplicates to the receiver, the receiver receives one of them first. In the receiver, only this first packet is used and the packets that arrived later are discarded. As described above, the receiver-side *MuTran* is relatively simple, so we do not describe the details.

### 3.4. Implementation

We implemented *MuTran* by modifying source files of MPTCP, specifically mptcp_sc hed.c, mptcp_ctrl.c, skbuff.c, mptcp.h, and skbuff.h [24]. After that, we implemented *MuTran* in Linux kernel of Ubuntu 16.04 LTS on computing boards for drones. (The computing board, Intel Aero compute board, performs a variety of computing operations, except for processing performed by the flight controller. The Intel Aero compute board is a UAV/Drone developer board powered by a quad-core Intel Atom x7-Z8750 processor [25].) We built a test-bed composed of two *MuTran*-implemented computing boards equipped with two network interfaces for performance evaluations. We will describe the detailed explanation about experiments in Section 5.

## 4. Theoretical Analysis

In this section, we conduct a theoretical analysis of the delay of control packet transmission when using *MuTran*. We then analyze practical cases with *MuTran* to demonstrate the validity of *MuTran*. After that, we figure out the number of subflows needed to guarantee the target delay, and we analyze the overhead due to the duplication. Furthermore, we conduct the analysis of control packet transmission rate when using *MuTran*. Table 1 lists the parameters and variables used in the analyses.

### 4.1. Element Delays of Control Packet Transmission

For this analysis, we assume a situation where there is one sender and one receiver and they have multiple identical interfaces. Each interface of the sender is connected to the corresponding interface of the receiver. These communication pairs utilize different channels to avoid interference. For example, in Figure 1, the number of pairs is two. The sender continues to send data packets to the receiver, while control packets are periodically created and transmitted. The order of control packets is separated from that of data packets. The delay means the time from one moment a packet is delivered to a sub-socket in the sender to the other moment a sub-socket in the receiver delivers the packet to the upper layer. In this analysis, we focus on the delay of only control packet transmission. Compared to data packets, control packets are generated much less frequently, and the time between consecutive transmissions is relatively long. Thus, reordering control packets rarely occurs, so we do not consider the delay caused by reordering control packets in this analysis.

When the data communications are in a steady-state, all the communications through subflows are identical. In this situation, the delay of *i*th subflow, $delay_{i}^{total}$, consists of three element delays as follows:

(1) $$delay_{i}^{total}=delay_{i}^{sq}+delay_{i}^{net}+delay_{i}^{rq},$$

where $delay_{i}^{sq}$, $delay_{i}^{net}$, and $delay_{i}^{rq}$ represent delays due to buffer queuing in the sender, propagation delay, and buffer queuing in the receiver, respectively.

### 4.2. Analysis of the Control Packet Transmission over TCP

The first element delay in Equation (1), $delay_{i}^{sq}$, is defined as follows:

(2) $$delay_{i}^{sq}=size_{i}^{sq}/rate_{i}^{send}.$$

The sending queue is usually filled with packets because the sender and receiver communicate without any restrictions. Therefore, if the sender’s queue size and the sending rate are $size_{i}^{sq}$ and $rate_{i}^{send}$, respectively, the time between when one control packet is inserted into the queue and when the packet is transferred from the sender is as shown in Equation (2).

If *W* and RTT represent the maximum size of congestion window and the round trip time in the steady-state, respectively, the congestion window size increases from *W*/2 to *W* and falls back to *W*/2 repeatedly [26]. Therefore, the relation between *W* and $rate_{i}^{send}$ can be expressed as follows:

(3) $$\begin{array}{cc} rate_{i}^{send} & =\left\{\left(3W^{2}/8\right)\cdot MSS\right\}/\left(RTT\cdot W/2\right)\\ \; & =3W\cdot MSS/4RTT, \end{array}$$

where MSS means the maximum segment size. In terms of *W*, Equation (3) can be transformed as follows:

(4) $$W=4rate_{i}^{send}\cdot RTT/3MSS.$$

By default, the initial size of the buffer in the network layer in the kernel is set to the default buffer size, and the operating system adjusts the size as needed. Taking account of the sending rate fluctuation, the operating system sets the send buffer size (The send buffer and receive buffer are terms used in Linux kernel.) to double the maximum amount of data that can be sent using one window as follows:

(5) $$\begin{array}{c} size_{i}^{sq}=2W\cdot MSS. \end{array}$$

Using Equation (4), Equation (5) can be transformed as follows:

(6) $$\begin{array}{c} size_{i}^{sq}=8rate_{i}^{send}\cdot RTT/3. \end{array}$$

Using Equation (6), Equation (2) can be transformed as follows:

(7) $$delay_{i}^{sq}=8RTT/3.$$

Secondly, since each interface of the sender is directly connected to the corresponding interface of the receiver, $delay_{i}^{net}$ in Equation (1) is defined as follows:

(8) $$delay_{i}^{net}=dist/c,$$

where *c* and dist respectively mean the speed of light and the distance between the sender and receiver.

The third element delay in Equation (1) is defined as follows:

(9) $$delay_{i}^{rq}=size_{i}^{rq}/rate_{i}^{proc},$$

where $size_{i}^{rq}$ and $rate_{i}^{proc}$ are the receiver’s queue size and the processing rate, respectively. Similar to the send buffer, the size of the receive buffer is set to double the value of the advertised window size multiplied by MSS. The communication between the sender and receiver in this situation is closed loop, so the amount of packets in the receiver’s receive buffer should be smaller than that of the sender’s send buffer. Therefore, the upper bound of $size_{i}^{rq}$ is $size_{i}^{sq}$ in Equation (6). As a result, according to Equation (9), the upper bound of $delay_{i}^{rq}$ is $8rate_{i}^{send}\cdot RTT/3rate_{i}^{proc}$. The rate at which the receiver processes packets is much faster than that at which the sender transmits packets. Therefore, in general, $delay_{i}^{rq}$ is much smaller than $delay_{i}^{sq}$.

### 4.3. Analysis of the Control Packet Transmission with Considering Retransmission

So far, we have considered delays without retransmissions. However, retransmissions can occur due to a variety of causes, so we should additionally consider delays caused by retransmissions. Every time a retransmission occurs, a lost packet is inserted to the send buffer and transmitted again, so $delay_{i}^{sq}+delay_{i}^{net}$ is added to $delay_{i}^{total}$. Thus, this addition is repeated continuously as many times as the number of retransmissions until the packet is successfully delivered. If *p* is the probability that a packet is transmitted successfully and *q*, (1 − *p*), is the probability of transmission failure, the probability that a packet is delivered successfully after *n* failed attempts is $q^{n}\cdot p$. Therefore, if $delay_{i}^{sq}+delay_{i}^{net}$ is simply expressed as $delay_{i}^{rt}$, the expected delay considering retransmissions, $E[delay_{i}^{total}]$, is as follows:

(10) $$\begin{array}{cc} E[delay_{i}^{total}] & =delay_{i}^{rq}+delay_{i}^{rt}\cdot p+2delay_{i}^{rt}\cdot qp+3delay_{i}^{rt}\cdot q^{2}p+\cdot \cdot \cdot \\ \; & =delay_{i}^{rq}+delay_{i}^{rt}\cdot p\sum_{k=1}^{\infty }kq^{k-1}\\ \; & =delay_{i}^{rq}+delay_{i}^{rt}\cdot \frac{p}{q}\sum_{k=1}^{\infty }kq^{k}. \end{array}$$

With reference to Appendix A, $\sum_{k=1}^{\infty }kq^{k}$ in Equation (10) can be transformed as follows:

(11) $$\sum_{k=1}^{\infty }kq^{k}=\lim_{n\to \infty }\sum_{k=1}^{n}kq^{k}=\lim_{n\to \infty }\frac{nq^{n+2}-\left(n+1\right)q^{n+1}+q}{{\left(q-1\right)}^{2}}.$$

Using Equation (11), Equation (10) can be transformed as follows:

(12) $$E\left[delay_{i}^{total}\right]=delay_{i}^{rq}+delay_{i}^{rt}\cdot \lim_{n\to \infty }\left\{\frac{p}{q}\cdot \frac{nq^{n+2}-\left(n+1\right)q^{n+1}+q}{{\left(q-1\right)}^{2}}\right\}.$$

(1 − *q*) is equal to *p*, so Equation (12) can be transformed as follows:

(13) $$E\left[delay_{i}^{total}\right]=delay_{i}^{rq}+delay_{i}^{rt}\cdot \lim_{n\to \infty }\frac{nq^{n+1}-\left(n+1\right)q^{n}+1}{p}.$$

The value of *q* is 0 to 1, so both $nq^{n+1}$ and $\left(n+1\right)q^{n}$ in Equation (13) become 0. Thus, Equation (13) can be transformed as follows:

(14) $$E\left[delay_{i}^{total}\right]=delay_{i}^{rq}+\frac{delay_{i}^{rt}}{p}.$$

When using *MuTran*, the control packet is duplicated, and the original and its duplicates are sent through different interfaces as shown in Figure 1d. If the number of interfaces that the sender or receiver has is *n*, the original packet and n−1 duplicate packets are transmitted via *n* interfaces. Therefore, the probability that at least one of them is delivered without failure, $p^{system}$, is as follows:

(15) $$\begin{array}{cc} p^{system} & =1-\left(1-p_{1}\right)\left(1-p_{2}\right)\cdot \cdot \cdot \left(1-p_{n}\right)\\ \; & =1-\prod_{i=1}^{n}\left(1-p_{i}\right), \end{array}$$

where $p_{i}$ is the probability that the transmission via the *i*th interface is successful. As we assumed, all the subflow communications are identical, so all $p_{i}$, *i* = 1, …, *n*, are the same. Thus, Equation (15) can be transformed as follows:

(16) $$\begin{array}{cc} p^{system} & =1-{\left(1-p\right)}^{n}\\ \; & =1-q^{n}. \end{array}$$

Based on Equations (14) and (16), the delay when using *MuTran* in TCP-mode with *n* interfaces is as follows:

(17) $$\begin{array}{cc} E[delay^{total}] & =delay^{rq}+\frac{delay^{rt}}{p^{system}}\\ \; & =delay^{rq}+\frac{delay^{rt}}{1-q^{n}}. \end{array}$$

According to Equation (17), even as *q* increases, the delay increases much slower, which means that *MuTran* enables users to control drones with short delays even in poor communication quality situations.

As explained before, $delay^{rq}\ll delay_{i}^{sq}<\left(delay_{i}^{sq}+delay_{i}^{net}\right)=delay^{rt}$, so Equation (17) can be simplified as follows:

(18) $$E\left[delay^{total}\right]\approx \frac{delay^{rt}}{1-q^{n}}.$$

**Figure 5:**
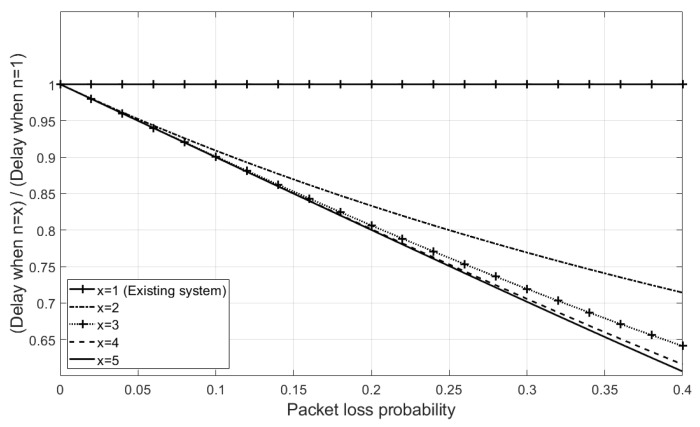
The ratio of delay when using *MuTran* in TCP-mode to that when using the existing system.

Based on Equation (18), Figure 5 shows the ratio of delay when using *MuTran* to that when using the existing system with one interface, as the packet loss probability increases.

For instance, if the packet loss probability is 0.25, *MuTran* with two interfaces can successfully transmit control packets within 80% of the delay when using the existing system on average. This means that, if an operator uses *MuTran*, the stability of controlling drones can be significantly improved simply by using one more interface.

### 4.4. Analysis of the Control Packet Transmission of MuTran in UDP-Mode

**Figure 6:**
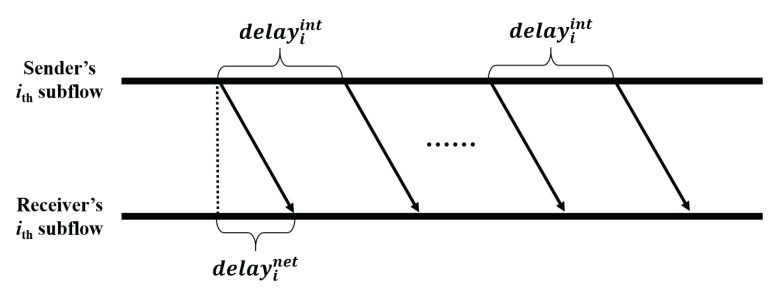
Periodical transmissions of control packet in the UDP mode.

In this subsection, we analyze the delay of control packet transmission of *MuTran* in UDP-mode. For comparison, we assume the same network situation as the situation considered in the previous subsection. In this situation, the sender transmits control packets with a determined time interval, $delay^{int}$, as shown in Figure 6, and $delay_{i}^{net}$ means the propagation delay.

If *p* is the probability that a packet is transmitted successfully and *q*, (1 − *p*), is the probability of transmission failure, the expected delay, $E[delay_{i}^{total}]$, is as follows:

(19) $$\begin{array}{cc} E[delay_{i}^{total}] & =delay_{i}^{net}\cdot p+\left(delay_{i}^{net}+delay_{i}^{int}\right)\cdot qp+\left(delay_{i}^{net}+2delay_{i}^{int}\right)\cdot q^{2}p+\cdot \cdot \cdot \\ \; & =delay_{i}^{net}\cdot \left(p+qp+q^{2}p+\cdot \cdot \cdot \right)+delay_{i}^{int}\cdot \left(qp+2q^{2}p+3q^{3}p+\cdot \cdot \cdot \right)\\ \; & =delay_{i}^{net}p\cdot \frac{1-q^{n}}{1-q}+delay_{i}^{int}\cdot qp\sum_{k=1}^{\infty }kq^{k-1}. \end{array}$$

Similar to the development from Equation (10) to Equation (14), Equation (19) can be transformed as follows:

(20) $$\begin{array}{cc} E[delay_{i}^{total}] & =delay_{i}^{net}\cdot \frac{p}{1-q}+delay_{i}^{int}\cdot \frac{q}{p}\\ \; & =delay_{i}^{net}+delay_{i}^{int}\cdot \frac{1-p}{p}. \end{array}$$

The original packet and n−1 duplicate packets are transmitted via *n* interfaces, and all the subflow communications are identical. Therefore, the probability that at least one of them is delivered without loss, $p^{system}$, is as follows:

(21) $$\begin{array}{cc} p^{system} & =1-{\left(1-p\right)}^{n}\\ \; & =1-q^{n}. \end{array}$$

As a result, Equation (20) can be transformed as follows:

(22) $$\begin{array}{cc} E[delay^{total}] & =delay^{net}+delay^{int}\cdot \frac{q^{n}}{1-q^{n}}. \end{array}$$

Since $delay^{net}$ is dist/c, $delay^{net}$ is much less than $delay^{int}$. Thus, Equation (22) can be simplified as follows:

(23) $$\begin{array}{c} E\left[delay^{total}\right]\approx delay^{int}\cdot \frac{q^{n}}{1-q^{n}}. \end{array}$$

**Figure 7:**
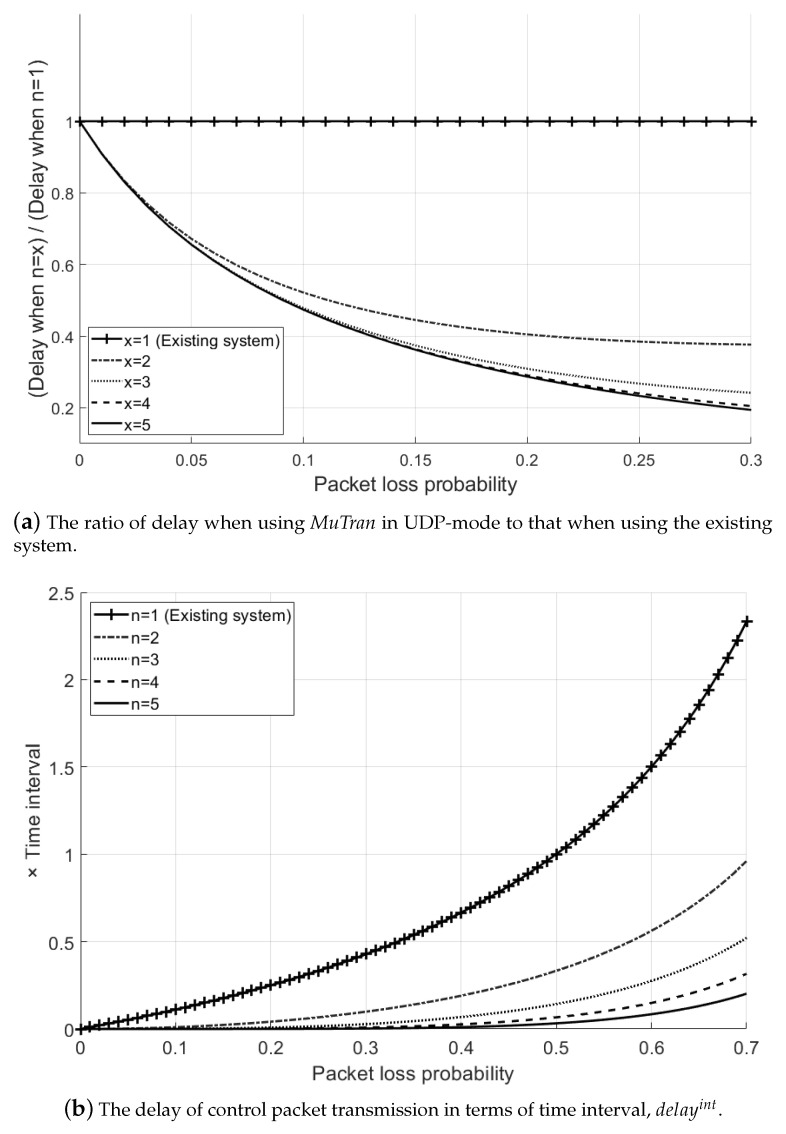
Analysis of the delay when using *MuTran* in UDP-mode.

Similar to Figure 5, based on Equation (23), Figure 7a shows the ratio of delay when using *MuTran* in UDP-mode to that when using the existing system with one interface, as the packet loss probability increases. When using UDP, a device does not conduct retransmission even when a packet loss occurs. Thus, when transmitting control packets over UDP, it is much effective to use *MuTran* which exponentially decreases the system probability of packet loss as shown in Figure 7a compared to the Figure 5.

In addition, Figure 7b shows the delay in terms of the value of time interval, $delay^{int}$, as the packet loss probability increases. As shown in the figure, by using the existing system, a control packet is delivered to a UAV after one more time interval when the packet loss probability is 0.5. In other words, during this time, the UAV flies without any control from the operator, which may cause an accident. On the other hand, in the same situation, a control packet can be delivered in a much lower delay by using *MuTran*.

### 4.5. Analysis of Practical Cases with MuTran

**Figure 8:**
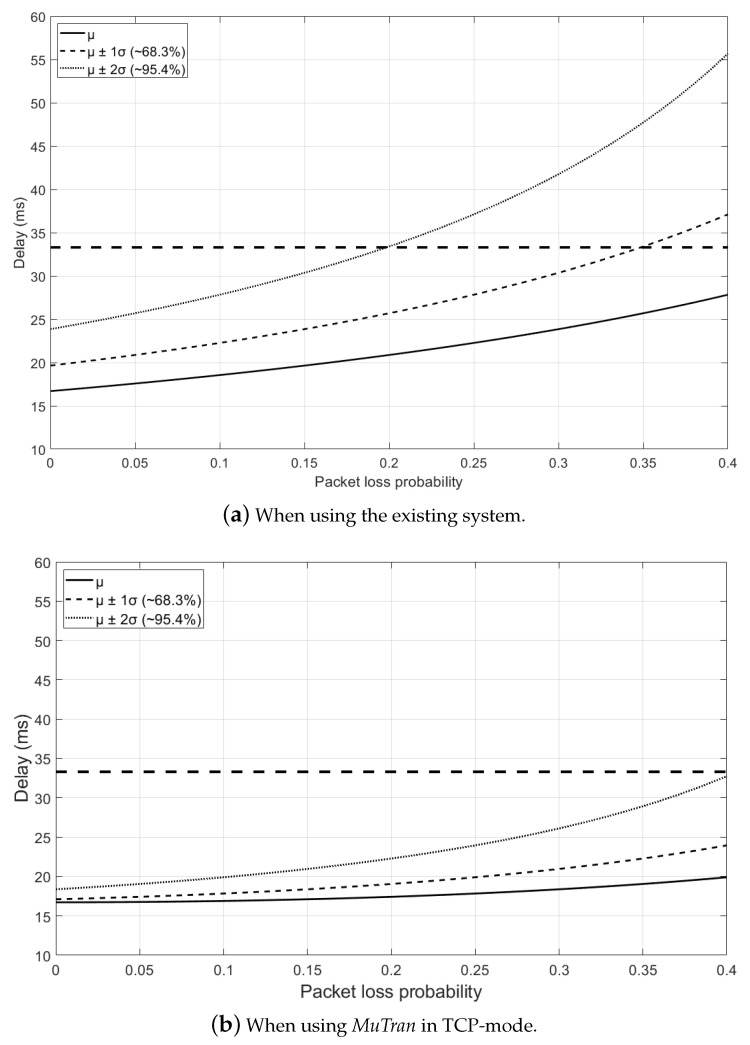

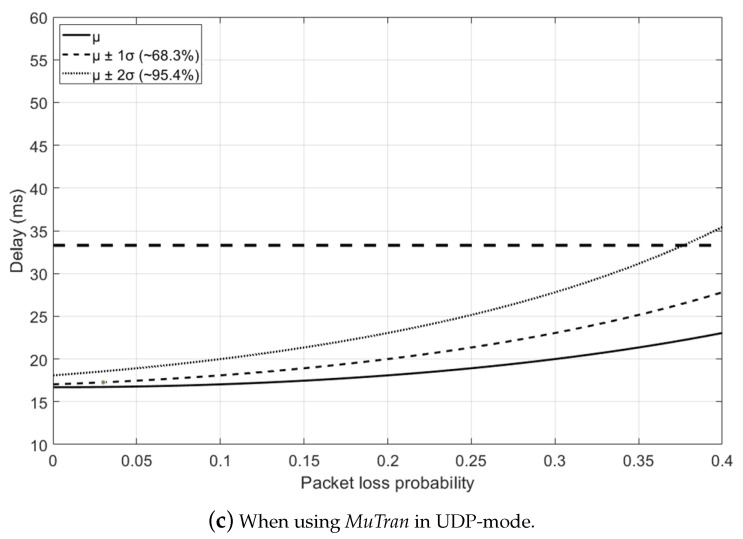
The analytic delay results in the practical cases.

To conduct an analysis of *MuTran* in practical situations, we assume that one drone is communicating with the ground control system (GCS) over a WiFi connection and that an operator controls the drone by viewing the video collected by the drone. Under this circumstance, if a 720p30 stream is encoded at 1 Mbps and the available bandwidth is 2 Mbps, it takes 118.7 ms for the operator to see the collected video [27]. In other words, if the drone is flying at a speed of 15 m/s, the drone will travel 1.8 m while the operator senses the need to change the flight and gives instructions. In addition to this, considering analysis results in [28,29] which analyzed drone networks experimentally, we analyze the delay of control packet transmission when using *MuTran* with two interfaces compared to the existing system. In the assumed circumstance, if the operator or GCS detects a problem from the *i*th packet transmitted from the drone, the operator or GCS immediately transmits the control packet to cope with the problem. To properly respond to the problem, the control packet should be delivered to the drone before the *i* + 1th packet arrives at the GCS. In other words, the target delay in this situation is the time between consecutive packet arrivals at the GCS. The dotted line in Figure 8 indicates the target delay.

According to Figure 8a which shows the result when using the existing system, if the packet loss probability is 0.2 or less, approximately 95% of packets can be delivered within the target delay. However, if the packet loss probability increases to 0.35, only about 68% percent of packets are delivered within the target delay. In contrast, using *MuTran* in TCP-mode, most of the packets can be delivered to the drone within the target delay as shown in Figure 8b, even when the packet loss probability is 0.4, which means the severe communication situation. Similarly, in Figure 8c, we can see that *MuTran* in UDP-mode is able to deliver most packets to the drone within the target delay even in poor communication situations.

### 4.6. Analysis of the Number of Subflows Required to Guarantee the Target Delay

As described above, *MuTran* allows much more control packets to be transferred within the target delay. Based on Equation (18), if Equation (24) is valid, it is guaranteed that control packets are delivered to the drone within the target delay in the TCP-mode.

(24) $$E\left[delay^{total}\right]\approx \;\frac{delay^{rt}}{1-q^{n}}=\frac{delay^{sq}+delay^{net}}{1-q^{n}}\leq delay^{target},$$

where $delay^{target}$ presents the target delay. Equation (24) can be transformed as follows:

(25) $$q^{n}\leq 1-\frac{delay^{sq}+delay^{net}}{delay^{target}},$$

and Equation (25) also can be transformed as follows:

(26) $$n\geq log_{q}\left(1-\frac{delay^{sq}+delay^{net}}{delay^{target}}\right).$$

Similarly, in the UDP-mode, if Equation (27) is valid based on Equation (23), control packets are guaranteed to be delivered to the drone within the target delay.

(27) $$E\left[delay^{total}\right]\approx delay^{int}\cdot \frac{q^{n}}{1-q^{n}}\leq delay^{target}.$$

(28) $$q^{n}\leq \frac{delay^{target}}{delay^{int}+delay^{target}}.$$

(29) $$n\geq log_{q}\left(\frac{delay^{target}}{delay^{int}+delay^{target}}\right).$$

Thus, using Equation (26) or (29) depending on the mode of *MuTran*, we can obtain the number of required interfaces to guarantee that 68%, 95%, or 99% of control packets are transmitted to the drone within the target delay by putting μ+σ, μ+2σ, or μ+3σ in $delay^{target}$, respectively.

### 4.7. Analysis of Overhead Due to Duplication in MuTran

Until now, we conducted the analyses of delays in *MuTran*, but in this subsection, we analyze the overhead due to duplication of control packets. The throughput of data packet transmission is rarely affected by *MuTran* since *MuTran* duplicates only control packets that are created less often and are smaller in size compared to data packets in general. However, when the network condition is so poor, the duplicated packets can be retransmitted over and over again, which may increase the overhead due to the duplication in *MuTran*. Similar to Equation (10), the expected total size of control packets without duplication, $E[size^{wodup}]$, is as follows:

(30) $$\begin{array}{cc} E[size^{wodup}] & =size^{cp}p+2size^{cp}qp+3size^{cp}q^{2}p+\cdot \cdot \cdot \\ \; & =size^{cp}p\cdot \sum_{k=1}^{\infty }kq^{k-1}\\ \; & =\frac{size^{cp}}{p}, \end{array}$$

where $size^{cp}$ presents the size of control packet. Based on Equation (30), with considering duplication in *MuTran* additionally, the expected total size of control packets when the number of connections is *n*, $E[size^{wdup}]$, is as follows:

(31) $$\begin{array}{cc} E[size^{wdup}] & =n\cdot E[size^{wodup}]\\ \; & =\frac{n\cdot size^{cp}}{p}. \end{array}$$

### 4.8. Analysis of Control Packet Transmission Rate When Using MuTran

We analyzed the expected delay and the expected total size of control packets when using *MuTran* in TCP or UDP-mode before. Thus, based on Equations (18) and (31), we can figure out the transmission rate of control packet when using *MuTran* in TCP-mode as follows:

(32) $$E\left[rate_{CP}^{TCP}\right]\approx \frac{n\cdot size^{cp}\left(1-q^{n}\right)}{delay^{rt}p}.$$

Similarly, based on Equations (23) and (31), the control packet transmission rate of *MuTran* in UDP-mode is as follows:

(33) $$\begin{array}{c} E\left[rate_{CP}^{UDP}\right]\approx \frac{n\cdot size^{cp}\left(1-q^{n}\right)}{delay^{int}pq^{n}}. \end{array}$$

When a packet loss occurs, *MuTran* in TCP-mode immediately retransmits the lost packet, whereas *MuTran* in UDP-mode sends the next control packet after the determined period of time regardless of the packet loss. Thus, if it is required to achieve as much reliability of control packet transmission as using TCP while using UDP, *MuTran* in UDP-mode transmits control packets more frequently, in other words, with a shorter time interval under the condition expressed in Equation (36).

(34) $$E\left[rate_{CP}^{UDP}\right]\leq E\left[rate_{CP}^{TCP}\right].$$

(35) $$\frac{nsize^{cp}\left(1-q^{n}\right)}{delay^{int}pq^{n}}\leq \frac{nsize^{cp}\left(1-q^{n}\right)}{delay^{rt}p}.$$

(36) $$\frac{delay^{rt}}{q^{n}}\leq delay^{int}.$$

## 5. Performance Evaluation

In this section, we describe the various experiments we conducted, and we show the results that evaluate *MuTran*’s performance. First, we describe an experiment that shows the limitations of existing systems. After that, we perform a performance evaluation comparing *MuTran* and the existing systems and show the results that *MuTran* is superior to the others.

### 5.1. Performance of Existing System in the Problem Situation

**Figure 9:**
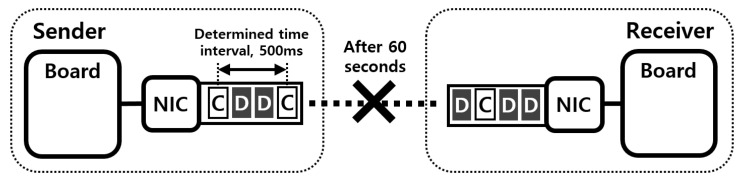
The experiment setup for evaluating the existing system in the problem situation.

In existing systems for drone networks, each drone has only one interface and communicates with other drones and with GCS over ad-hoc networks. Therefore, if the communication quality of the only interface deteriorates, drones have trouble in receiving control packets from the GCS. The loss of connection means the loss of control over the drone, and the delayed transmission or loss of control packets can cause catastrophic accidents. We conducted an experiment to confirm this problem. As shown in Figure 9, in this experiment, there were two computing boards for drones, Intel Aero compute boards, one sender and one receiver, and each board had one IEEE 802.11 g interface. After the experiment began, the sender transmitted data and control packets to the receiver. The data packets were continuously sent to the recipient, while the control packets were created and transmitted with a determined time interval, 500 ms. After 60 s, we removed the sender’s antenna to emulate a situation in which the quality of the communication link deteriorates. During the experiment, we recorded moments when the receiver received control packets and time intervals between consecutive moments.

**Figure 10:**
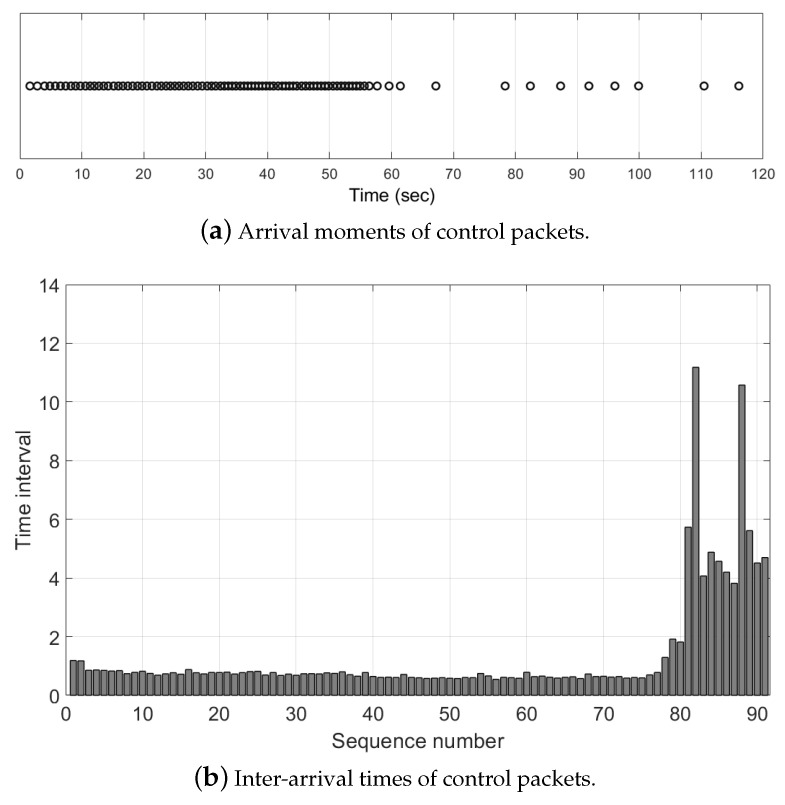
The limitation of existing system in the problem situation.

Figure 10a shows the arrival moments of control packets, and we can see that the receiver received control packets periodically early in the experiment. However, after one minute, the delayed transmissions or losses of control packets occurred frequently due to poor communication conditions, so the receiver received the control packets occasionally. More specifically, Figure 10b shows the inter-arrival times of control packets. At the beginning of the experiment, there was no significant difference between inter-arrival times as shown in the figure. However, after the communication quality deteriorated, inter-arrival time values were increased significantly. As shown in these results, it is hard to deal with these communication problem situations with the existing systems.

### 5.2. Evaluation of Transmitting Packets in a Round-Robin Fashion

**Figure 11:**
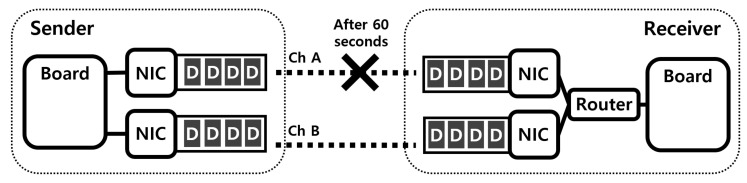
The experiment setup for evaluating packet transmissions in a round-robin fashion. (In order to use MPTCP, it is required that the routing tables in the sender and receiver are configured appropriately and the multiple interfaces are connected to the receiver’s board via a router [30].)

As explained in Section 3.2, the straightforward way to transfer the original and duplicate packets over different paths is to transfer them in a round-robin fashion using multiple interfaces. However, the overall performance of data communication is degraded because the sender using this method transmits packets without considering the communication situation. We compared performances of MPTCP’s two schedulers, the round-robin scheduler and the default scheduler that selects the subflow with the shortest SRTT. Similar to the previous experiment, there were two boards for drones, one sender and one receiver. However, in this experiment, each board had two interfaces, and each interface of the sender was connected to the corresponding interface of the receiver as shown in Figure 11. The sender and receiver communicated via two paths, and these communication pairs used different channels to avoid interference. The sender began sending packets to the receiver, and after 60 s, we made the communication quality poor by removing the sender’s antenna.

**Figure 12:**
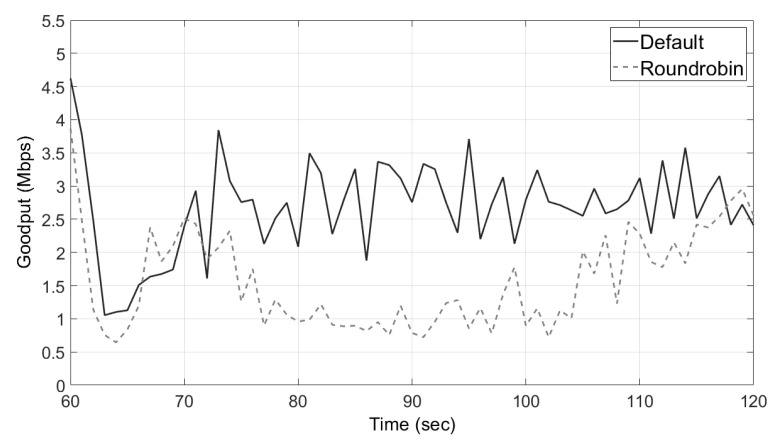
The round-robin scheduler’s problem in a low-quality communication situation.

Figure 12 shows the performances of two schedulers in a low-quality communication situation. As shown in the figure, the round-robin scheduler failed to respond to the change in the communication situation, while the default scheduler performed data communication appropriately through the interface with better communication quality. Thus, the default scheduler outperformed the round-robin scheduler, which means that the straightforward way explained in the previous paragraph is not suitable for *MuTran*. For this reason, we designed *MuTran* to be compatible with the default scheduler as explained in Section 3.3.1.

### 5.3. Evaluation on the Performance of MuTran

**Figure 13:**
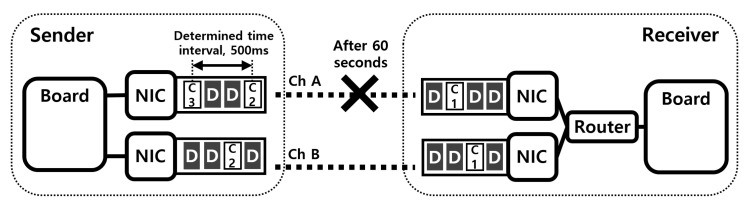
The experiment setup for evaluating the performance of *MuTran*

We conducted an experiment to validate the effectiveness of *MuTran*. Similar to the experiment in Section 5.2, the sender started data communications through two paths, and after 60 s, we deteriorated the communication quality of one path by removing the sender’s antenna. However, unlike the previous experiment, the sender transmitted not only data packets but also control packets as shown in Figure 13. The data packets were delivered continuously to the receiver, but the control packets were transmitted at a fixed time interval, 500 ms. The sender duplicated every control packet and then transmitted the original and its duplicate to the receiver.

**Figure 14:**
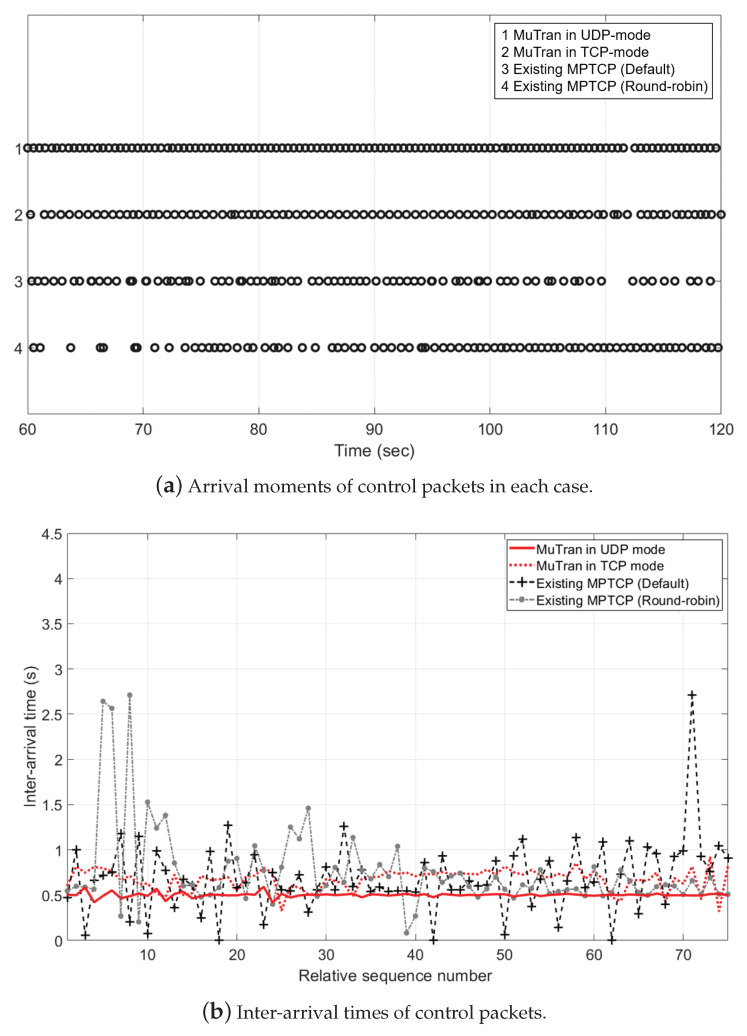
Performance comparison among *MuTran* and existing systems.

We compared four cases in this experiment. The first and second cases are when using *MuTran* in UDP-mode and TCP-mode, respectively. In the third and fourth cases, the sender simply duplicated control packets and transmitted them using the existing system with MPTCP, so it was not guaranteed that the original and its duplicate were transmitted through different paths. The default scheduler and the round-robin scheduler were used in the third and fourth cases, respectively. When the communication quality was good, there was no problem with data communication naturally. Thus, Figure 14 shows only the results after the communication condition deteriorated.

Figure 14a shows the arrival moments of control packets. As shown in the figure, in the first and second cases using *MuTran*, the rings are almost uniformly placed, which means that delayed transmissions or losses of control packets rarely occurred. In contrast, delayed transmissions or losses of control packets frequently occurred in the third and fourth cases, so distances between consecutive points are considerably different. To be more specific about the second case, there were some delayed transmissions, in other words, there are some overlapping circles. It is because that data and control packets are inserted into the same network queue when using *MuTran* in TCP-mode. Thus, the latency of control packet transmission in *MuTran*’s TCP-mode can be occasionally lengthened due to diverse causes, such as the increase in the amount of data packets or data packet retransmissions. However, in UDP-mode, *MuTran* transmits control packets through UDP connections separated by TCP connections used for the data transmission, so it is possible to transfer control packets with much less fluctuation as shown in the first case.

Figure 14b shows the results in terms of inter-arrival times of control packets. Compared to the results of the first and second cases, there are much more fluctuations in the results of the third and fourth cases, which results in the unstable control of drones. In addition, Table 2 shows the mean and standard deviation values of inter-arrival times in each case. There is no significant difference between the standard deviation values of the cases when the communication was in a normal situation. However, when the communication quality was poor, the values of the third and fourth cases are much larger than those of the first and second cases. Especially, the values of the first case are much lower than those of the other cases because control packets were transmitted through UDP connections not affected by data packet transmissions, as explained in the previous paragraph.

In conclusion, through the results of experiments, we confirm that *MuTran* improves the reliability and stability of controlling drones.

### 5.4. Performance Comparison with Related Techniques

**Figure 15:**
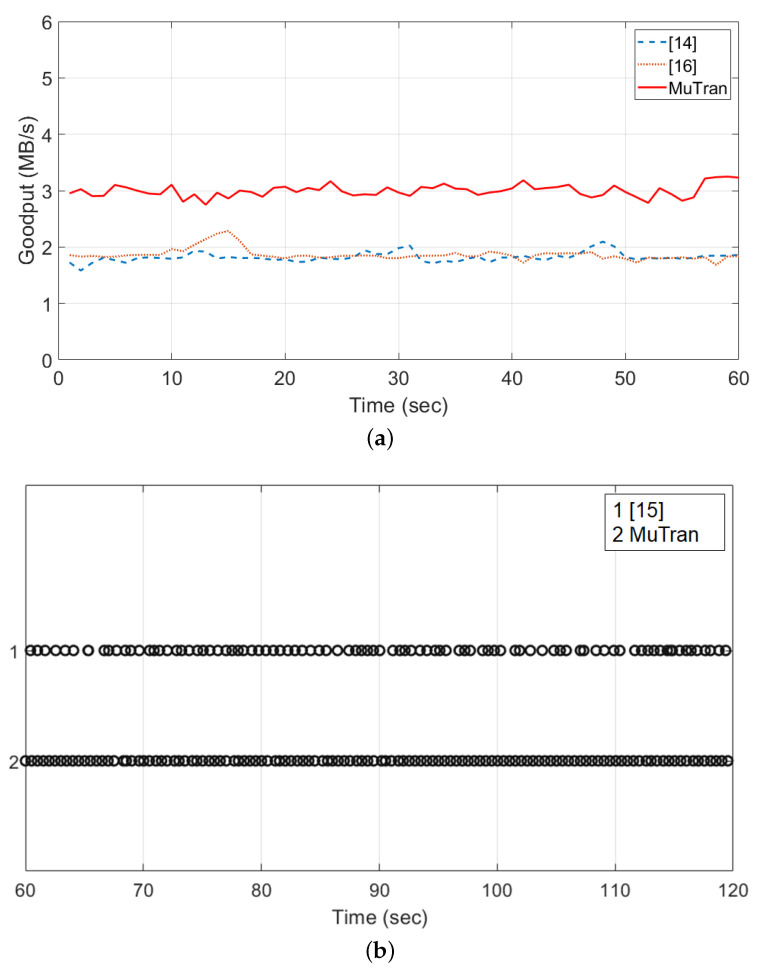
The comparison between the related techniques and *MuTran*. (**a**) The goodput performances of the data packet transmission of the related techniques [14,16] and *MuTran*. (**b**) Arrival moments of control packets in a low-quality communication situation when using the related technique [15] and *MuTran*.

In Section 2, we introduced some techniques relevant to *MuTran* and explained the limitations of them. The technique proposed in [14] copies all packets, data and control packets, and transmits them over two different paths, so this technique unnecessarily wastes half the communication bandwidth. Another technique in [16] assigns data packets and control packets separately to two different interfaces, so the interface for control packet cannot be used to transmit data packets. Therefore, these techniques cannot enhance the goodput of data packet transmission even with two interfaces. To verify this, we conducted the experiment by imitating the techniques with reference to the descriptions in their papers. In this experiment, we conducted data communications via two interfaces similar to the setup in Figure 13 and measured goodput performances for 1 min. As explained above, the related techniques performed about half of *MuTran*’s performance in Figure 15a.

The other related technique in [15] replicates only control packets to improve [14]. However, data and control packets are inserted into the same queue competitively, so the latency of control packet transmission can be affected by data packet transmissions. To prove the limitation, we conducted the same experiment as we did in the previous section, and Figure 15b shows the arrival moments of control packets. As shown in the figure, the distances between consecutive points are inconsistent when using the related technique. In other words, the latency of control packet transmission was occasionally lengthened because the data and control packets were competitively inserted into the same network queue. The distribution of circles are also not perfectly uniform when using *MuTran*. It is because the network interface was shared although UDP and TCP transmissions used separated queues in their respective connections. However, as shown in the figure, the interference by data packet transmissions was considerably isolated because of transmitting control packets separately.

## 6. Discussion

In this section, we describe several issues that need to be addressed further. Firstly, we explain the complexity and feasibility of *MuTran* and then discuss *MuTran* in UAV-based scenarios.

### 6.1. Complexity and Feasibility of MuTran

As explained in Section 3.3, *MuTran* conducts the three major operations, the classification, duplication, and assignment, so we analyze the complexity and overhead of the operations.

The classification is required for *MuTran* to duplicate only control packets, not data packets. In the kernel layer, it is not trivial to know which data contains control information, so the process for checking the content of each packet is added when using *MuTran*. *MuTran* directly reads segment data of each packet by using the pointer that points to the starting position of segment in memory. In this process, if *MuTran* looks through all the segment data, it may cause a large amount of computation. However, *MuTran* only checks some values in front part of the segment data to determine the characteristic of packets. Most control protocols for UAV systems, such as MAVLink, use control packets that include their own header format [21]. Thus, *MuTran* does not examine all the segment, but only looks over a few bytes of segment to determine whether this part follows the header format of control packet. In addition, *MuTran* utilizes the pointer in struct sk_buff, built in Linux kernel, so *MuTran* is able to approach to the segment in memory almost without overhead.

*MuTran* duplicates control packets only. Thus, when *MuTran* is used with *n* interfaces, the total size of packets is as follows:

(37) $$packet^{total}=packet^{data}+n\cdot packet^{control}.$$

Control packets are created less and relatively small compared to data packets in general. In addition to this, in real-world scenarios, the drone is not equipped with a large number of network interfaces considering the efficiency and the drone’s limited resources. Therefore, the process of duplicating control packets does not cause much overhead. However, the proportion of $n\cdot packet^{control}$ in $packet^{total}$ may increase depending on the application or situation. Thus, if the second term is not negligible, the operator needs to consider the overhead based on Equation (37).

*MuTran* conducts the assignment operation to forward duplicated packets to different interfaces. Fundamentally in Linux kernel, the function, get_available_subflow(), is repeatedly called to find the best subflow among available subflows according to the rule, such as the shortest SRTT. For example, if the selection is based on the shortest SRTT, the function calculates the SRTT value of each subflow and returns the pointer of socket with the shortest SRTT when *MuTran* is not used. However, when using *MuTran*, the function returns the best one of sockets belonging to the interfaces which are unused to transmit duplicated packets. Thus, *MuTran* utilizes values that are already calculated regardless of the use of *MuTran*, and the assignment operation causes very little additional computation.

As a result, *MuTran* does not require much computation and the complexity of *MuTran* is not high, so *MuTran* can be applied to UAV systems without much overhead in real-world scenarios. However, as we mentioned above, the amount of computations for *MuTran*’s operations can increase depending on the application or situation. Thus, we will perform a deeper analysis of complexity and feasibility of *MuTran* in various applications and real-world scenarios in the future work.

### 6.2. Applicability of MuTran in UAV-Based Scenarios

*MuTran* is proposed to improve the reliability and stability of controlling UAVs. Thus, in this subsection, we explain various features of UAV-based scenarios. After that, we describe the advantages and effects of *MuTran* from the perspective of the features.

#### 6.2.1. Physical Aspects in Aerial Scenarios

UAVs can fly fast and move freely, so there are several different features in aerial scenarios compared to ground-based scenarios. Firstly, in terms of the distance, flying UAVs are usually sparse with long distances between UAVs. Thus, weak signals due to such long distances make communications of UAVs unstable [28]. The exploitation of multiple antennas might be limited by the lack of spatial diversity of the aerial UAV-to-UAV (U2U) channel [29]. In addition, the communication quality can be degraded by the Doppler effect owing to the relative difference in velocity between flying UAVs. The Doppler shift, $\frac{f_{c}\cdot \Delta v}{c}$, caused by the UAVs’ relative motion may result in packet errors [31]. $f_{c}$, Δv, and *c* mean the frequency, the relative speed, and the light speed, respectively. Moreover, the antenna’s orientation and position on the UAV also affect the communication quality. UAVs fly in a 3-dimensional space. Therefore, the signal propagation is almost always partially obstructed by a UAV’s body and the relative orientation of antennas exists at all times, which deteriorates the communication quality [32]. Taken together, due to the diverse factors explained above, the communications in aerial scenarios are generally much more unstable than those in ground-based scenarios. Thus, *MuTran* can be utilized effectively to improve the communication reliability in UAV-based scenarios.

#### 6.2.2. High Mobility and Frequent Topology Change

The high mobility is a representative feature of UAVs, and this is an important characteristic that differentiates UAV network, such as flying ad hoc networks (FANETs) from other similar networks, such as mobile ad hoc networks (MANETs) or vehicular ad hoc networks (VANETs). The status of UAVs, such as position, velocity, acceleration, flight plan, and so forth, varies over time since they often have to react to the environmental feedback and mission updates [33]. In addition, according to the nature of flying, UAVs must be sparse apart in the sky with large distances between them [34]. Such high mobility degree of UAVs results in several communication and networking issues and challenges [35]. In UAV-based scenarios, UAVs may change their places, and distances between UAVs vary in a much shorter time interval. Thus, the network topology changes frequently, which affects the link quality between UAVs and increases the likelihood that the communication problems occur. In addition to this, the lack of a fixed topology poses a great challenge to the routing process in UAV scenarios. For example, discovered routes frequently break and new routes should be discovered. Thus, compared to other systems, UAV systems require more reliable and robust control message transmissions to cope with the above problems well. As a result, *MuTran* is more effective in such systems composed of UAVs with high mobility.

#### 6.2.3. A Wide Range of Networks and Communications

To perform various tasks and services, flying UAVs should be able to communicate with each other, U2U, as well as with backbones, UAV-to-Infrastructure (U2I) [35]. In addition, there are various networks used in UAV scenarios, such as Ad-Hoc networks, cellular networks, delay-tolerant networks, and satellite networks. Thus, many different communication and network techniques can be available for UAVs, such as IEEE 802.11 (WiFi), IEEE 802.16 (WiMax), LTE/5G, and satellite communications [36,37]. These techniques have different uses and characteristics in terms of radio frequency, modulation, MAC, data rate, transmission range, and so forth, so they are more effective when used together. Thus, by utilizing heterogeneous networks simultaneously, UAVs not only perform more diverse tasks and services, but also improve the performance such as increased bandwidth and enhanced reliability. Since *MuTran* utilizes different paths and protocols, *MuTran* can be applied to UAVs with heterogeneous interfaces well. Furthermore, if *MuTran* transmits duplicated control packets using heterogeneous interfaces as well as different paths and protocols, the reliability and stability of controlling UAVs can be improved much more.

#### 6.2.4. Diverse Applications and Systems Using UAVs

From an operational point of view, UAV systems have a wide range of applications, and there are various types of traffic. For example, some UAV applications may need to transmit real-time traffic in contrast to other applications with delay-tolerant traffic. *MuTran* was proposed to deliver control packets in time without loss or delay in this paper, but *MuTran* with some modifications can also be effectively used to transmit time-constrained data packets. In some use cases, multiple UAVs are used together to accomplish given missions. For example, in agriculture, several UAVs could work together to effectively spray large fields with pesticides or to quickly distribute the seeds over large areas [35]. In such cases, it is important to provide the robust and reliable connectivity among UAVs in the system, so *MuTran* can be utilized to improve the stability of the UAV systems.

## 7. Conclusions

Today’s UAVs can perform various operations while flying, and because of many advantages of UAVs, they are utilized in various fields. Since UAVs move freely in any direction and fly fast, controlling UAVs reliably is paramount to operate UAVs. In particular, the UAV’s fast movement and wireless communication’s instability increase the loss or delay of the control packet transmission, which increases the likelihood of a collision or crash. For this reason, in this paper, we proposed *MuTran* which improves the reliability of UAV control by transmitting duplicated control packets utilizing multiple protocols and paths. We designed and implemented *MuTran* on real devices. We performed various experiments, as well as theoretical analyses from various perspectives, to verify the effectiveness of *MuTran*. Through the analyses and experiments, we confirmed that, by using *MuTran*, control packets can be delivered to UAVs more stably, which improves the reliability of controlling UAVs.

We have several directions as future work. We plan to improve *MuTran* to selectively duplicate control packets by considering the transmission failure rate rather than duplicating all control packets without any condition. Furthermore, as we mentioned in Section 6, we will perform a deeper analysis of complexity and feasibility of *MuTran* in real-world scenarios and evaluate the applicability of *MuTran* in various UAV-based scenarios. We also have a plan to enhance *MuTran*’s ability to leverage heterogeneous networks to control UAVs more reliably by exploiting different network interfaces simultaneously.

## Figures and Tables

**Table 1:** The parameters and variables used in the analyses.

Notation	Definition
*W*	The maximum size of congestion window in the steady-state
RTT	The round trip time in the steady-state
MSS	The maximum segment size
*c*	The speed of light
dist	The distance between the sender and receiver
*p*	The probability of transmission success
*q*	The probability of transmission failure
$delay_{i}^{total}$	The total delay of *i*th subflow
$delay_{i}^{sq}$	The buffer queuing delay of *i*th subflow in the sender
$delay_{i}^{rq}$	The buffer queuing delay of *i*th subflow in the receiver
$delay_{i}^{net}$	The propagation delay of *i*th subflow
$delay_{i}^{rt}$	The sum of propagation delay and buffer queuing delay of *i*th subflow in the sender, ($delay_{i}^{net}+delay_{i}^{sq})$
$delay^{int}$	The determined time interval for control packet transmission
$delay^{target}$	The target delay to meet the required service quality
$size_{i}^{sq}$	The queue size of *i*th subflow in the sender
$size_{i}^{rq}$	The queue size of *i*th subflow in the receiver
$size^{cp}$	The size of control packet
$size^{wodup}$	The total size of control packets without duplication
$size^{wdup}$	The total size of control packets with duplication
$rate_{i}^{proc}$	The processing rate of *i*th subflow in the receiver
$rate_{i}^{send}$	The sending rate of *i*th subflow in the sender
$rate_{CP}^{TCP}$	The control packet transmission rate in TCP-mode
$rate_{CP}^{UDP}$	The control packet transmission rate in UDP-mode

**Table 2:** Mean and standard deviation of inter-arrival times of control packets.

	Normal Situation		Problematic Situation	
Case	Mean	Std	Mean	Std
1	0.5002	0.0027	0.5046	0.0511
2	0.5849	0.1155	0.6591	0.1665
3	0.6095	0.142	0.8929	0.6721
4	0.5879	0.1638	0.6993	0.4067

## Data Availability

Data sharing not applicable.

## Abbreviations

The following abbreviations are used in this manuscript:

UAV	Unmanned Aerial Vehicle
TCP	Transmission Control Protocol
MPTCP	Multipath Transmission Control Protocol
UDP	User Datagram Protocol
MAC	Media Access Control
SDN	Software Defined Network
LTE	Long Term Evolution
RC	Radio Controller
MIMO	Multiple Input Multiple Output
MPR	Multi-Packet Reception
HRI	Human-Robot Interaction
FEC	Forward Error Correction
SRTT	Smoothed Round Trip Time
API	Application Programming Interface
GCS	Ground Control System
U2U	UAV to UAV
U2I	UAV to Infrastructure
FANET	Flying Ad hoc Network
MANET	Mobile Ad hoc Network
VANET	Vehicular Ad hoc Network

## Appendix A

(A1) $$\sum_{i=1}^{n}i\cdot a^{i}=a+2a^{2}+3a^{3}+\cdot \cdot \cdot +na^{n}.$$

Adding $na^{n+1}$ to both sides of Equation (A1) results in

(A2) $$\sum_{i=1}^{n}i\cdot a^{i}+na^{n+1}=a+2a^{2}+3a^{3}+\cdot \cdot \cdot +na^{n}+na^{n+1}.$$

Multiplying both sides of Equation (A1) by *a* results in

(A3) $$a\sum_{i=1}^{n}i\cdot a^{i}=a^{2}+2a^{3}+3a^{4}+\cdot \cdot \cdot +na^{n+1}.$$

Subtracting Equation (A3) from Equation (A2) results in

(A4) $$\begin{array}{cc} \sum_{i=1}^{n}i\cdot a^{i}-a\sum_{i=1}^{n}i\cdot a^{i}+na^{n+1} & =a+a^{2}+a^{3}+\cdot \cdot \cdot +a^{n}\\ \; & =\sum_{i=1}^{n}a^{i}\\ \; & =\frac{a(a^{n}-1)}{a-1}. \end{array}$$

Subtracting $na^{n+1}$ from both sides of Equation (A4) results in

(A5) $$\sum_{i=1}^{n}i\cdot a^{i}-a\sum_{i=1}^{n}i\cdot a^{i}=\frac{a(a^{n}-1)}{a-1}-na^{n+1}.$$

Let $\sum_{i=1}^{n}i\cdot a^{i}=X$, and Equation (A5) can be transformed as follows:

$$\begin{array}{cc} X-aX & =\frac{a(a^{n}-1)}{a-1}-na^{n+1}\\ X(1-a) & =\frac{a(a^{n}-1)}{a-1}-na^{n+1}\\ X & =\frac{na^{n+1}}{a-1}-\frac{a(a^{n}-1)}{{\left(a-1\right)}^{2}}\\ X & =\frac{na^{n+2}-na^{n+1}}{{\left(a-1\right)}^{2}}-\frac{a(a^{n}-1)}{{\left(a-1\right)}^{2}} \end{array}$$

(A6) $$\begin{array}{cc} X & =\frac{na^{n+2}-na^{n+1}-a^{n+1}+a}{{\left(a-1\right)}^{2}}\\ X & =\frac{na^{n+2}-\left(n+1\right)a^{n+1}+a}{{\left(a-1\right)}^{2}}. \end{array}$$

We let $\sum_{i=1}^{n}i\cdot a^{i}=X$ before. Thus, Equation (A6) can be transformed as follows:

(A7) $$\sum_{i=1}^{n}i\cdot a^{i}=\frac{na^{n+2}-\left(n+1\right)a^{n+1}+a}{{\left(a-1\right)}^{2}}.$$
